# A Method To Prevent SARS-CoV-2 IgM False Positives in Gold Immunochromatography and Enzyme-Linked Immunosorbent Assays

**DOI:** 10.1128/JCM.00375-20

**Published:** 2020-05-26

**Authors:** Qiang Wang, Qin Du, Bin Guo, Daiyong Mu, Xiaolan Lu, Qiang Ma, Yangliu Guo, Li Fang, Bing Zhang, Guoyuan Zhang, Xiaolan Guo

**Affiliations:** a Department of Laboratory Medicine, Affiliated Hospital of North Sichuan Medical College, Nanchong, Sichuan, People’s Republic of China; b Faculty of Laboratory Medicine, North Sichuan Medical College, Nanchong, Sichuan, People’s Republic of China; c Center for Translational Medicine, North Sichuan Medical College, Nanchong, Sichuan, People’s Republic of China; d Department of Laboratory Medicine, Nanchong Central Hospital, Nanchong, Sichuan, People’s Republic of China; Boston Children's Hospital

**Keywords:** novel coronavirus, gold immunochromatography assay, enzyme-linked immunosorbent assay, false-positive, urea

## Abstract

We set out to investigate the interference factors that led to false-positive novel severe acute respiratory syndrome coronavirus 2 (SARS-CoV-2) IgM detection results using gold immunochromatography assay (GICA) and enzyme-linked immunosorbent assay (ELISA) and the corresponding solutions. GICA and ELISA were used to detect SARS-CoV-2 IgM in 86 serum samples, including 5 influenza A virus (Flu A) IgM-positive sera, 5 influenza B virus (Flu B) IgM-positive sera, 5 Mycoplasma pneumoniae IgM-positive sera, 5 Legionella pneumophila IgM-positive sera, 6 sera of HIV infection patients, 36 rheumatoid factor IgM (RF-IgM)-positive sera, 5 sera from hypertensive patients, 5 sera from diabetes mellitus patients, and 14 sera from novel coronavirus infection disease 19 (COVID-19) patients. The interference factors causing false-positive reactivity with the two methods were analyzed, and the urea dissociation test was employed to dissociate the SARS-CoV-2 IgM-positive serum using the best dissociation concentration. The two methods detected positive SARS-CoV-2 IgM in 22 mid-to-high-level-RF-IgM-positive sera and 14 sera from COVID-19 patients; the other 50 sera were negative. At a urea dissociation concentration of 6 mol/liter, SARS-CoV-2 IgM results were positive in 1 mid-to-high-level-RF-IgM-positive serum and in 14 COVID-19 patient sera detected using GICA. At a urea dissociation concentration of 4 mol/liter and with affinity index (AI) levels lower than 0.371 set to negative, SARS-CoV-2 IgM results were positive in 3 mid-to-high-level-RF-IgM-positive sera and in 14 COVID-19 patient sera detected using ELISA. The presence of RF-IgM at mid-to-high levels could lead to false-positive reactivity of SARS-CoV-2 IgM detected using GICA and ELISA, and urea dissociation tests would be helpful in reducing SARS-CoV-2 IgM false-positive results.

## Abstract (precis)

We set out to investigate the interference factors that led to false-positive novel severe acute respiratory syndrome coronavirus 2 (SARS-CoV-2) IgM detection results using gold immunochromatography assay (GICA) and enzyme-linked immunosorbent assay (ELISA) and the corresponding solutions. GICA and ELISA were used to detect SARS-CoV-2 IgM in 86 serum samples, including 5 influenza A virus (Flu A) IgM-positive sera, 5 influenza B virus (Flu B) IgM-positive sera, 5 Mycoplasma pneumoniae IgM-positive sera, 5 Legionella pneumophila IgM-positive sera, 6 sera of HIV infection patients, 36 rheumatoid factor IgM (RF-IgM)-positive sera, 5 sera from hypertensive patients, 5 sera from diabetes mellitus patients, and 14 sera from novel coronavirus infection disease 19 (COVID-19) patients.

## INTRODUCTION

The outbreak of infections by a novel coronavirus (nCoV) strain, severe acute respiratory syndrome coronavirus 2 (SARS-CoV-2), that began in Wuhan, China, has spread rapidly throughout the country, as well as to other countries around the world. The outlook of prevention and control of novel coronavirus infection disease 19 (COVID-19) is still grim. As of 20 February 2020, the number of confirmed COVID-19 cases exceeded 70,000, and this number has continued to rise steadily, placing enormous emphasis on the timely and accurate diagnosis and treatment of the disease. At present, diagnosis of COVID-19 is mainly based on epidemiological history inquiry, laboratory testing, and chest radiology examination. Among these examinations, detection of nucleic acid from SARS-CoV-2 represents direct evidence for COVID-19 diagnosis (1–3). Detection of SARS-CoV-2 nucleic acid should be performed in special laboratories by professional technicians, and the assays involved have the disadvantages of insufficient supply of detection kits in a public health emergency, low throughput, and time-consuming procedures. Moreover, the swabs taken from the throat may not always reveal the infection of SARS-CoV-2 for patients; additional sampling is always performed for accurate diagnosis. Therefore, nucleic acid detection may not the best choice for screening large-scale populations infected with SARS-CoV-2 (4).

The detection of serum-specific IgM and IgG, especially the former, is routinely used in clinical laboratories to evaluate the acute phase of infection of pathogens in the serum (5, 6). In many infections, IgM can be detected as early as 1 week after infection. When the level of IgM reaches the detection limit of the assay kit, the detection of IgM can avoid false-negative results owing to sampling. At present, the main methods for the detection of specific antibodies in clinical laboratories are gold immunochromatography assay (GICA) and enzyme-linked immunosorbent assay (ELISA) (7–11), both of which have the advantages of mature methodology, high flux detection, simple operation, rapid detection, lack of the need for special equipment, and low cost. Using these two methods to detect SARS-CoV-2 IgM can identify or screen SARS-CoV-2 infection in suspicious and close-contact populations earlier and more quickly and effectively and can improve the accuracy of epidemiological monitoring, which is very important for patient management and epidemic prevention and control. However, in the process of using GICA and ELISA to detect SARS-CoV-2 IgM, we found that there was interference from rheumatoid factor IgM (RF-IgM) in assays using the two methods.

The affinity of cross-reactions between specific antigens and antibodies was lower than that of specific reactions (12). Urea can be used as a substance for dissociation between antigen-antibody reactions to evaluate the affinity of IgG, such as the evaluation of the affinity of Toxoplasma gondii IgG in different detection systems (13, 14). Therefore, we hypothesize that the use of the urea dissociation test will help to eliminate or reduce the influence of RF-IgM on the detection of SARS-CoV-2 IgM antibodies. Meanwhile, IgM-positive sera of other pathogens were collected to evaluate the detection performance of GICA and ELISA for SARS-CoV-2 IgM.

## MATERIALS AND METHODS

### Study setting and patients.

This study was approved by the Ethics Committee of Affiliated Hospital of North Sichuan Medical College. Serum from a total of 86 patients with different pathogen infections and related chronic diseases were collected from the Affiliated Hospital of North Sichuan Medical College and Nanchong Central Hospital from 25 January 2020 to 15 February 2020. In accordance with the Notice on the Issuance of Strategic Guidelines for Diagnosis and Treatment of Novel Coronavirus (SARS-CoV-2) Infected Pneumonia (15), 5 patients with influenza A virus (Flu A) IgM-positive sera, 5 patients with influenza B virus (Flu B) IgM-positive sera, 5 patients with Mycoplasma pneumoniae IgM-positive sera, 5 patients with Legionella pneumophila IgM-positive sera, 6 patients with HIV infection, 36 patients with RF-IgM-positive sera, 5 hypertensive patients, and 5 diabetes mellitus patients had no clinical symptoms or imaging evidence of COVID-19. The other 14 (COVID-19) patients met the diagnostic criteria, and sera were collected within 3 to 7 days after the beginning of the clinical symptoms. In addition to the 36 RF-IgM-positive serum samples, detection levels of RF-IgM in the remaining 50 serum samples were lower than 20.00 IU/ml.

### Assay.

IgM against Flu A and Flu B, M. pneumoniae, and L. pneumophila was detected by indirect immunofluorescence assay (Respiratory tract 8 joint detection kit; EUROIMMUN, Inc., Germany). RF-IgM was detected by rate nephelometry assay (IMMAGE800, Beckman Coulter, Inc., USA). HIV combi pertussis toxin) (PT) was detected by electrochemiluminescence assay (Cobas E602; Roche, Inc., Germany). HIV infection was confirmed by immunoblotting assay (the confirmed information was fed back by CDC). SARS-CoV-2 nucleic acid was detected using real-time PCR (RT-PCR) (kit provided by Shanghai Zhijiang Biotechnology Co., Shanghai, China; detection instrument provided by Shanghai Hongshi Biotechnology Co., Shanghai, China). GICA and ELISA were used for SARS-CoV-2 IgM detection (kit provided by Beijing Hotgen Biotechnology Co., Beijing, China: lot no. 20200208 and 20200229 for GICA and lot no. 20200101 and 20200201 for ELISA). Optical density in ELISA plates was measured using a microplate reader (PHOmo; Autobio Diagnostics Co., Zhengzhou, China).

### Urea dissociation test of GICA.

Sera (100 μl) were added into 1-ml sample diluents (phosphate-buffered saline [PBS], NaCl, and Tween 20) and mixed, and then 100 μl of the diluted sample was put into the sample hole of the test card. The liquid was chromatographed upward under the control of the capillary effect; when the liquid was about to reach the upper absorbent paper, 100 μl PBS solution containing 6 mol/liter urea was added into the sample hole of the test card; the results were observed after 20 to 25 min. The SARS-CoV-2 IgM in the sample bound first with the anti-human-IgM labeled by colloidal gold and then with the SARS-CoV-2 recombinant antigen at the test line (T) position to form a complex of SARS-CoV-2 antigen, SARS-CoV-2 IgM, and anti-human IgM labeled by colloidal gold. A complex of goat polyclonal IgG and anti-human IgM labeled by colloidal gold was formed at the control line (C) position. For the positive standard, colloidal gold color reactions occur at both the T-line and C-line positions; for the negative standard, the colloidal gold color reaction occurs only at the C-line position.

### Urea dissociation test of ELISA.

Sera (8 μl) were added into 800-μl sample diluents (0.02 M PBS) and mixed, and then 100-μl volumes of the diluted sample, the negative control, and the positive control were added to the wells of plates coated with SARS-CoV-2 recombinant antigen, and the plates were incubated at 37°C for 30 min. The plates were washed five times, and 100 μl of PBS solution (containing 0 mol/liter, 1 mol/liter, 2 mol/liter, 4 mol/liter, 6 mol/liter, and 8 mol/liter urea in different wells) was added and incubated at 37°C for 10 min. After three more washes, anti-human IgM horseradish peroxidase (HRP)-labeled antibody was added into the reaction system to form an indirect immune complex. Following five washes to remove unbound substances, the substrate was added for the color reaction. The results were interpreted according to the ratios of the sample optical density value and the cutoff optical density value (S/CO) as follows: positive, S/CO equal to or greater than 1.00; negative, S/CO less than 1.00. The results of affinity index (AI) analyses were expressed as the ratios of the S/CO values determined for different dissociated urea concentrations to that of PBS with 0 mol/liter urea. The AI threshold value was set as the middle value between the highest AI value determined for the false-positive sample results with the outliers removed and the lowest AI value determined for all of the SARS-CoV-2 infection samples. The results were interpreted as follows: positive, AI value of sera greater than or equal to the AI threshold; negative, AI value of sera less than the AI threshold.

### Statistical analysis.

Statistical analyses were performed by the use of SPSS, version 19.0 (SPSS Inc., USA). Fisher’s exact test was used for the specific comparisons of GICA and ELISA results obtained for the detection of SARS-CoV-2 IgM in sera with positive RF-IgM before and after urea dissociation. The specific comparisons between the GICA and ELISA results obtained for the detection of SARS-CoV-2 IgM before and after urea dissociation in all control sera were made using Pearson’s chi-square test. The statistical significance of all tests was defined as a *P* value of <0.05 determined by two-tailed tests.

## RESULTS

### Results of analyses of 2019 nCoV IgM in different serum samples.

SARS-CoV-2 IgM results were negative in both GICA and ELISA for the 5 Flu A IgM-positive sera, 5 Flu B IgM-positive sera, 5 M. pneumoniae IgM-positive sera, 5 L. pneumophila IgM-positive sera, 6 sera from HIV infection patients, 5 sera from hypertensive patients, and 5 sera from diabetes mellitus patients (Table 1). A total of 22 of the 36 RF-IgM-positive samples were positive, and 14 COVID-19 patient samples were positive for SARS-CoV-2 IgM in both GICA and ELISA (Table 1; see also Table 2).

**TABLE 1:** SARS-CoV-2 IgM detection in serum using GICA and ELISA

Group	No. of cases	No. (%) of positive cases	
		GICA	ELISA
Flu A IgM	5	0 (0.00)	0 (0.00)
Flu B IgM	5	0 (0.00)	0 (0.00)
M. pneumoniae IgM	5	0 (0.00)	0 (0.00)
L. pneumophila IgM	5	0 (0.00)	0 (0.00)
Hypertension	5	0 (0.00)	0 (0.00)
Diabetes mellitus	5	0 (0.00)	0 (0.00)
HIV infection	6	0 (0.00)	0 (0.00)
RF-IgM	36	22 (61.11)	22 (61.11)
SARS-CoV-2 infection	14	14 (100.00)	14 (100.00)

**TABLE 2:** RF-IgM-positive serum results of SARS-CoV-2 IgM detected using GICA and ELISA^*a*^

Patient no.	RF-IgM (IU/ml)	GICA	ELISA
1	34.2	N	N
2	35.5	N	N
3	39.8	N	N
4	56.2	N	N
5	56.3	N	N
6	66.5	N	N
7	69.2	N	N
8	73.6	N	N
9	74.2	P	P
10	76.2	P	P
11	82.8	P	P
12	84.0	N	N
13	98.80	N	N
14	118.0	P	P
15	119	P	P
16	142	P	P
17	145	P	P
18	154	P	P
19	161	P	P
20	173	P	P
21	173	P	P
22	195	N	N
23	220	N	N
24	222	P	P
25	224	P	P
26	256	P	P
27	283	P	P
28	284	N	N
29	328	P	P
30	431	P	P
31	440	P	P
32	441	N	N
33	502	P	P
34	1,050	P	P
35	1,680	P	P
36	1,680	P	P

a N, negative; P, positive.

### Comparison of SARS-CoV-2 IgM results and detection performance before and after urea dissociation test of GICA.

**FIG 1:**
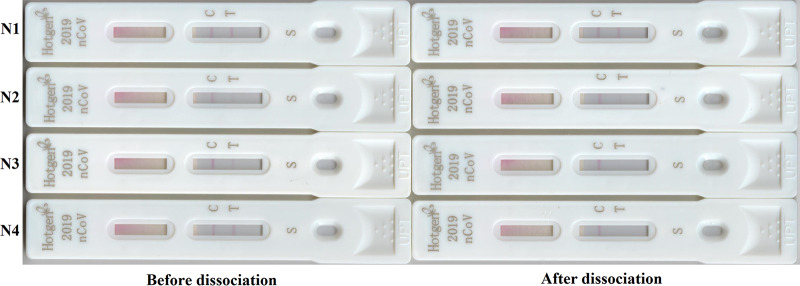
SARS-CoV-2 IgM detected using GICA before and after urea dissociation. N1 and N2, SARS-CoV-2 IgM in serum of SARS-CoV-2 infection patients before and after urea dissociation test in GICA; N3 and N4, SARS-CoV-2 IgM in serum with RF-IgM-positive results before and after urea dissociation test in GICA.

When the dissociation concentration of urea was 6 mol/liter, the dissociation test of GICA was carried out for 22 sera with RF-IgM positive results and 14 samples from COVID-19 patients that were positive for SARS-CoV-2 IgM in GICA before urea dissociation. The results of SARS-CoV-2 IgM analysis performed for 21 serum samples with positive RF-IgM turned negative (Fig. 1), whereas those for the 14 samples from the COVID-19 patients remained positive. In the urea dissociation test, the specificity of GICA after dissociation was significantly higher than before dissociation (*P* < 0.001), and the sensitivity was not affected (Table 3).

**TABLE 3:** Comparison of specificities of GICA SARS-CoV-2 IgM detection before and after urea dissociation

Control	No. of cases	% specificity (no. of cases showing indicated level of GICA specificity/total no. of cases)	
		Before dissociation	After dissociation
RF-IgM negative	36	100.00 (36/36)	100.00 (36/36)
RF-IgM positive	36	38.89 (14/36)	97.22 (35/36)^*a*^

Total	72	69.44 (50/72)	98.61 (71/72)^*a*^

a *P *< 0.001 (compared with results determined before dissociation).

### Comparison of SARS-CoV-2 IgM results and detection performance before and after urea dissociation test of ELISA.

**FIG 2:**
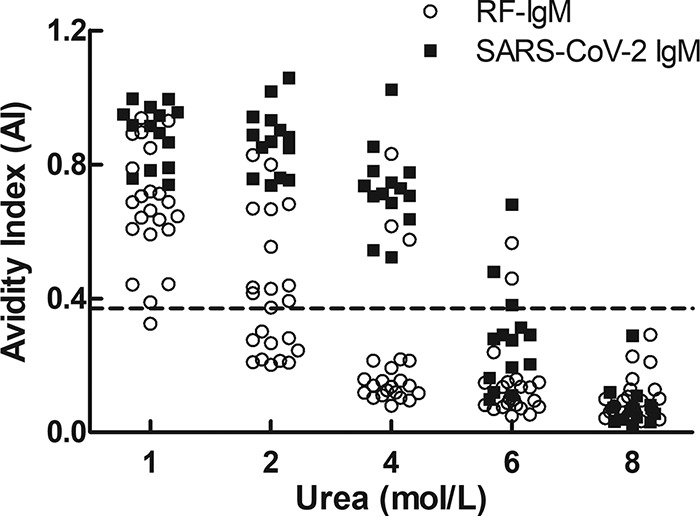
AI of SARS-CoV-2 IgM detected using different urea dissociation concentrations of ELISA. When the dissociation concentration of urea was 4 mol/liter and the AI calculation method value was set to 0.371, the results determined for SARS-CoV-2 IgM in 19 sera with RF-IgM positivity turned negative, whereas the results determined for SARS-CoV-2 IgM in the 14 sera from COVID-19 patients remained positive.

The urea dissociation test of ELISA was carried out with PBS containing 0 mol/liter, 1 mol/liter, 2 mol/liter, 4 mol/liter, 6 mol/liter, and 8 mol/liter urea in 22 RF-IgM-positive serum samples and serum from 14 COVID-19 patients that were positive for SARS-CoV-2 IgM in ELISA before urea dissociation. When the dissociation concentration of urea was 4 mol/liter and with the AI calculation method set to 0.371, the results of SARS-CoV-2 IgM analyses in 19 serum samples with positive RF-IgM turned negative, whereas those of SARS-CoV-2 IgM in 14 serum samples from COVID-19 patients remained positive (Fig. 2). Through the urea dissociation test, the specificity of ELISA after dissociation was found to be significantly higher than that before dissociation (*P* < 0.001), and the sensitivity was unaffected (Table 4).

**TABLE 4:** Comparison of specificities of ELISA SARS-CoV-2 IgM detection before and after urea dissociation

Control	No. of cases	% specificity (no. of cases showing indicated level of ELISA specificity/total no. of cases)	
		Before dissociation	After dissociation
RF-IgM negative	36	100.00 (36/36)	100.00 (36/36)
RF-IgM positive	36	38.89 (14/36)	91.67 (33/36)^*a*^

Total	72	69.44 (50/72)	95.83 (69/72)^*a*^

a *P *< 0.001 (compared with results determined before dissociation).

## DISCUSSION

SARS-CoV-2 infection patients have many clinical symptoms similar to those of patients infected with common respiratory tract pathogens such as Flu A, Flu B, M. pneumoniae, and L. pneumophila, including fever, fatigue, and cough. Moreover, the majority of COVID-19 patients have had preexisting diseases such as diabetes, hypertension, and other endocrine and metabolic diseases (16, 17). Therefore, we fully considered the situation described above in selecting the control population for the present study. According to some reports, the bloodwork of SARS-CoV-2 infection patients mainly showed decreased lymphocyte counts. Therefore, this study also included HIV infection patients with similar phenomena in the control group (1, 2, 17). The number of patients enrolled in each group was limited owing to insufficient amounts of diagnostic reagents and available time. At the same time, before the completion of the trial, only 14 cases of COVID-19 patients were recruited in our study. Although the relatively small sample size inevitably shows some bias in these two methods, the improvement in specificity was clear.

RF is an autoantibody against the FC segment of denatured IgG, the main type of which is IgM. It is the main factor that causes the interference in immune responses (18–20). When the SARS-CoV-2 IgM test was carried out for all control serum and serum from COVID-19 patients by GICA and ELISA, the results showed that false-positive interference occurred only in RF-IgM-positive serum, and the serum results from COVID-19 patients were all positive, indicating that the two methods had high sensitivity but that their specificity needed to be improved. The results of this study showed that when the RF-IgM concentration was lower than 70 IU/ml, there was no interference between the two methods in detecting SARS-CoV-2 IgM. In the other 29 sera with a RF-IgM concentration above 70 IU/ml, 22 cases showed positive results from the two methods, suggesting that the presence of a mid-to-high level of RF-IgM greatly influenced the detection of SARS-CoV-2 IgM. However, the results of SARS-CoV-2 IgM detection assays in the other seven sera with a high RF-IgM level were negative, which may have been related to the blocking of the cross-reaction site of RF-IgM and needs further investigation. The explanation for the mechanism that caused RF-IgM false-positive results in the two methods may be that RF-IgM reacts with SARS-CoV-2 recombinant antigen and that RF-IgM combines with gold-labeled anti-human IgM or HRP-labeled anti-human IgM, giving false-positive results. This cross-reactivity can be reduced by urea dissociation.

A mid-to-high level of RF-IgM can cause false-positive results of SARS-CoV-2 IgM detection by GICA and ELISA. Therefore, when serum is RF-IgM positive, it is difficult to determine the real SARS-CoV-2 IgM status. This study attempted to eliminate or reduce the interference by urea dissociation. The main rationale for the selection of the time point used for addition of urea solution in the GICA method was as follows: first, after a certain reaction time, the specific antigen antibody may bond more firmly, thus making the procedure of urea dissociation more challenging; second, at that time point, the amount of liquid content in the sample well was small, with little change in urea concentration in the dissociation solution added later, thereby ensuring the dissociation effect. According to our previous study (21), the urea dissociation concentration in GICA was 6 mol/liter, with the results for 21 of the 22 RF-IgM-positive sera that had given false-positive SARS-CoV-2 IgM results turning negative, whereas the 14 serum samples from COVID-19 patients were not affected. In addition, when the urea dissociation concentration in ELISA was 4 mol/liter and the dissociation time was 10 min, the results for 19 of the 22 with RF-IgM-positive sera that had given false-positive results for SARS-CoV-2 IgM turned negative, whereas the 14 sera from COVID-19 patients were not affected. Therefore, the improved GICA and ELISA outcomes not only ensured detection sensitivity but also improved the corresponding specificity and reliability. The ELISA urea dissociation test showed detection performance similar to that of the GICA urea dissociation test, and this may have resulted from the use of the same recombinant antigen and the fact that both methods are based on the same detection principle.

In conclusion, when GICA and ELISA are used to detect SARS-CoV-2 IgM, the level of RF-IgM in the serum should be evaluated, and the urea dissociation test should be carried out to avoid the risk of false-positive results. At the same time, the results of this study suggest that the urea dissociation test cannot completely eliminate interference from RF-IgM. Therefore, when SARS-CoV-2 IgM results are still positive after urea dissociation, RT-PCR should be used for nucleic acid detection. In addition, it should be emphasized that serological tests represent a method that is complementary to nucleic acid detection. The preferred method for detection of acute disease is via molecular testing, rather than testing for IgM, precisely because of the possibility of inaccurate results. On the basis of our research results, we suggest that all of the methods mentioned above should be used to eliminate or reduce the impact of cross-reaction when using GICA and ELISA methods to detect SARS-CoV-2 IgM, which will help in the preliminary screening of suspected and high-risk groups, as well as in the assessment, prevention, and control of the SARS-CoV-2 epidemic and the formulation of appropriate prevention systems.
